# Protection Against *Salmonella* by Vaccination with Toxin–Antitoxin Self-Destructive Bacteria

**DOI:** 10.3390/vaccines14010089

**Published:** 2026-01-15

**Authors:** Nady Gruzdev, Jacob Pitcovski, Chen Katz, Nili Ruimi, Dalia Eliahu, Caroline Noach, Ella Rosenzweig, Avner Finger, Ehud Shahar

**Affiliations:** 1 MIGAL Galilee Research Institute, Kiryat Shmona 11016, Israel; 2 The Faculty of Life Sciences, Tel-Hai Academic College, Upper Galilee 12208, Israel; 3 Phibro Animal Health, Bet Shemesh 9905503, Israel

**Keywords:** *Salmonella*, toxin–antitoxin, Hok/Sok, CeaB/CeiB, FNR, PhoP

## Abstract

**Background**: *Salmonella* is a major zoonotic foodborne pathogen. Conventional poultry vaccines may present limitations in terms of efficacy, safety, and practicality. **Objectives**: This study focuses on enhancing the immunogenicity and improving the safety of a novel oral vaccination employing inducible toxin–antitoxin (TA) systems, which lead to self-destruction of virulent *Salmonella* Enteritidis. **Methods**: A Hok/Sok (HS) TA system was designed to induce cell death upon absence of arabinose. Point mutations were introduced to the Hok toxin promoter to moderate toxin production. A combination of HS and CeaB/CeiB (CC) TA systems was designed to induce cell death both in low di-cation levels or anaerobic conditions. Survival of *Salmonella*-carrying TA systems was tested in culture and in the Raw264.7 macrophage cell line. One-day old chicks were inoculated with *Salmonella* carrying the TA system to evaluate bacterial persistence and induction of a protective immune response. **Results**: Attenuation of the Hok toxin promoter prolonged bacterial survival in vitro. *Salmonella* carrying the combined TA systems was eliminated completely both in vitro and in inoculated chickens, eliciting high levels of antibodies and conferring protection against challenge with wild-type *Salmonella*. **Conclusions**: These findings highlight the potential of the adaptable TA-based vaccination platform to generate safe and efficacious *Salmonella* vaccines for poultry, contributing to reduced transmission in the food chain.

## 1. Introduction

*Salmonella* is a zoonotic foodborne pathogen that colonizes the intestinal tract of multiple hosts, including mammals, avians, and reptiles. Each year, there are an estimated 1.3 billion cases of non-typhoid salmonellosis, with 3 million deaths throughout the world [1]. The most common sources of foodborne *Salmonella* infection in human are poultry eggs and meat; therefore, prevention of human infection is mostly achieved by vaccination of farm animals [2,3]. The *Salmonella* vaccines for poultry include both inactivated and live attenuated vaccines. Inactivated vaccines are administered either intramuscularly or subcutaneously and require at least two immunizations. These vaccines stimulate the short-term production of high levels of antibodies, eliciting relatively low cell-mediated and mucosal immunity [4]. In contrast, live attenuated vaccines induce antibody- and cell-mediated immune responses to a broad repertoire of antigens. However, live attenuated vaccines carry risks of reversion to virulence and possible interference with *Salmonella* diagnostics procedures [5]. Furthermore, the attenuated bacteria strain may persist for long periods in chickens and their surrounding environment [6,7]. In our previous study [8], we developed a novel, orally administered, anti-*Salmonella* vaccination strategy that combined the effectiveness of live-attenuated vaccines with the safety of inactivated vaccines. The approach involved engineering of an inducible self-destructive toxin–antitoxin (TA) system integrated into the bacterial vaccine strain. In their natural bacterial hosts, these systems play an important role in cellular persistence, virulence, stress adaptation, biofilm formation, and stable maintenance of exogenous DNA during cell division [9,10]. TA systems are generally composed of a stable toxin that reduces membrane integrity or mRNA stability or disrupts essential cellular processes, such as DNA replication or protein synthesis, alongside a labile antitoxin that inhibits the deleterious activity of the toxin [11]. Two of the most studied TA systems in the Enterobacteriaceae family are Hok/Sok (HS) and CeaB/CeiB (CC). HS drives a type I post-segregational killing mechanism encoded by the R1 plasmid in *E. coli*. Hok is a small stable toxic protein that causes cell death by depolarization of the cell membrane, while the Sok gene encodes complementary RNA, forming a duplex with the leader region of Hok mRNA, which is then recognized by RNase III and degraded [12,13].

Colicin E2 (ColE2) is a bacteriocin system encoded by the pBRE2/ColE2 plasmid in *E. coli*. The ColE2 system comprises a secreted DNAase, CeaB, and its neutralizing protein, CeiB, which directly binds and subsequently inactivates it, protecting the producing bacteria [14].

In our previous study, four synthetic TA systems based on HS or CC, activated by either host-dependent or host-independent conditions, were designed and tested for safety and immunogenicity both in vitro and in a chicken model [8]. In the host-independent approach, the toxin was coupled to a strong constitutive promoter, and the antitoxin was coupled to an arabinose-inducible promoter (pBAD). In the absence of arabinose, expression of antitoxin was minimal, and the cells were killed by the constitutively expressed toxin. The host-dependent approach exploited host conditions specific to Salmonella infection, such as a low-oxygen environment of the intestine during bacterial attachment and low abundance of Mg^2+^ and Ca^2+^, following penetration into epithelial cells [15]

Fumarate and nitrate reductase (FNR) is a pivotal regulator protein involved in O_2_ sensing in *Salmonella* [16,17]. In order to activate cell death induced by the transition of *Salmonella* from an aerobic to an anaerobic environment, we coupled the CeaB toxin to the ansB promoter enhanced by FNR and the CeiB antitoxin to a FNR-repressed promoter cyoA [18]. Similarly, we coupled the Hok/Sok TA system to mgtL/A and treR promoters regulated by the pleiotropic two-component system PhoP, aiming to activate cell death in low concentrations of Mg^2+^ and Ca^2+^, conditions that are prevalent following bacterial penetration into epithelial cells [19].

Under restrictive conditions, the designed TA induction systems caused significant bacterial killing in transformed *E. coli* and *S.* Enteritidis in vitro. Inoculation of chickens with TA-carrying *Salmonella* resulted in undetectable bacteria levels in the gastrointestinal (GI) tract by day 10. Notably, while chicks vaccinated with *Salmonella* carrying the FNR-CC or the PhoP-HS TA systems developed a robust immune response, low bacterial levels were still detected in the liver by day 10, raising some safety concerns. In contrast, bacteria carrying the pBAD-HS TA system seemed to best fulfill safety requirements, with complete elimination of the vaccine bacteria in less than 3 days, but it failed to induce a significant immune response [8].

In the present study, a series of modified TA systems were developed to optimize the oral delivery of live bacteria to enable their survival for a controlled period and enhance the induction of immune responses and protection.

## 2. Material and Methods

### 2.1. Bacterial Strains and Growth Conditions

*Salmonella enterica*, *enterica* serovar Enteritidis strain B3 (Phibro, Beit Shemesh, Israel) cultures were prepared by incubating an inoculation of a single colony in 5 mL Luria broth (LB) overnight (O.N.) under permissive conditions (Table 1). Thereafter, bacteria were transferred to restrictive conditions (see Table 1) for 24 h. Bacterial suspensions were then serially diluted, plated on LB agar, and further incubated O.N. under permissive conditions before colony forming units (CFUs) were quantitated.

### 2.2. Construction of Modified TA Systems

**Figure 1:**
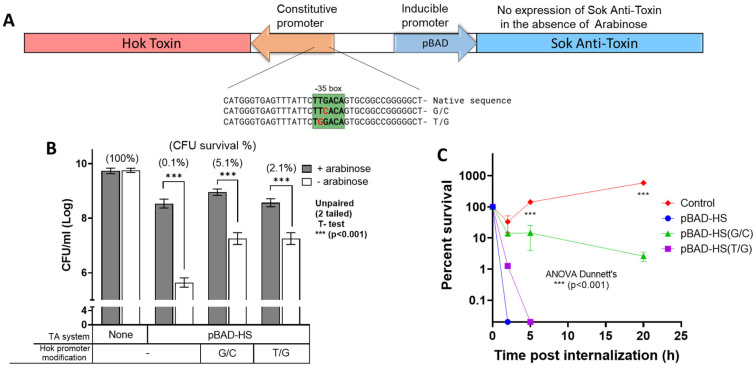
**Effects of attenuation of the constitutive Hok toxin promoter on bacterial survival under restrictive conditions and within macrophages.** (**A**). Hok expression was attenuated by either G/C or T/G substitution (marked in red) at the promoter’s −35 consensus sequence (highlighted in green) (**B**). *Salmonella* transformants carrying pBAD-HS with the native promoter sequence or with attenuated promoters were grown for 24 h under restrictive conditions, as described in the Materials and Methods Section. *Salmonella* carrying an empty pBR322 plasmid served as a control. Bars represent mean colony forming unit (CFU) counts per ml of three independent experiments, each of which was performed with triplicate samples. The y axis is presented in log scale. The percentage reduction of CFU in restrictive versus permissive conditions is indicated in brackets above each relevant bar. (**C**). Survival of *Salmonella* wild-type strain and strains carrying pBAD-HS systems was measured following uptake by macrophages, as described in the Materials and Methods Section. Percent survival is presented in log scale and was calculated as the number of CFUs at various time points post-internalization, as compared to baseline CFU counts. Graphs represent values from two independent experiments, each of which was performed with triplicate samples.

The TA systems used for this study were designed based on previously reported constructs [8]. As illustrated in Figure 1, Hok toxin in the pBAD systems were alternatively synthesized, coupled to promoters containing single nucleotide replacements predicted to yield attenuated protein production [20].

**Figure 2:**
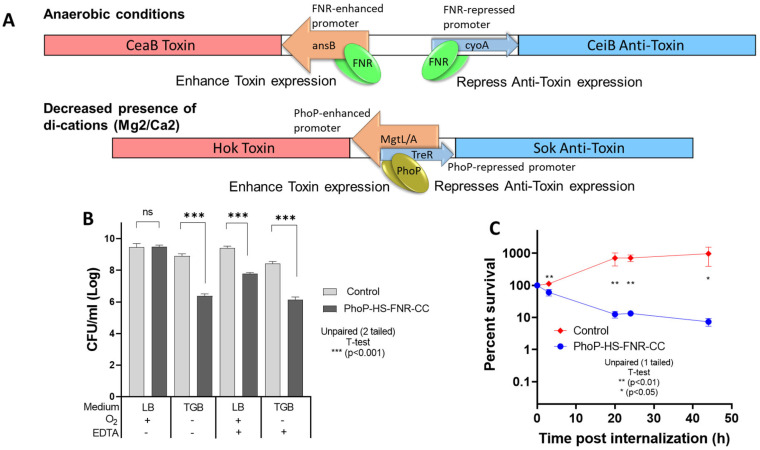
**Survival of PhoP-HS-FNR-CC-carrying *Salmonella* under restrictive conditions and within macrophages.** (**A**). Design of FNR and PhoP toxin–antitoxin activation systems. (**B**) *Salmonella* transformants carrying the PhoP-HS-FNR-CC system were grown for 24 h under restrictive conditions, as described in the Materials and Methods Section. Strains carrying an empty pBR322 plasmid served as a control. Bars represent mean percentage of survival in three independent experiments, each of which was performed with triplicate samples. The y axis is presented in log scale. (**C**). Survival of *Salmonella* wild-type and strains carrying the PhoP-HS-FNR-CC system was measured following infection of macrophages, as described in the Materials and Methods Section. Percent survival is presented in log scale and was calculated as the number of CFUs at various time points post-internalization, as compared to baseline CFU counts. Graphs represent values from two independent experiments, each of which was performed with triplicate samples.

The modified pBAD-HS constructs were cloned into a pBR322 vector between SalI and EcoRI restriction sites. The combined FNR-CC and PhoP-HS system (Figure 2A) was generated by introducing PhoP-HS into the SalI cloning site (underlined) of a pBR322 vector carrying FNR-CC using CGCGTCGACGGGCTACCGGCGAACCAGCA and CGCGTCGACGCTTGAGGCTTTCTGCCTCA primers. The plasmids were transformed into *S*. Enteritidis using standard methods, and positive clones were selected on LB agar plates supplemented with ampicillin (100 µg/mL) and further verified by polymerase chain reaction (PCR) and sequencing.

### 2.3. Survival Following Invasion into Macrophages

Mouse macrophage cell line Raw 264.7 (ATCC, Rockville, MD, USA) was grown in RPMI 141 medium supplemented with 10% fetal bovine serum (Biological Industries, Beit Haemek, Israel). Cultures of *S.* Enteritidis harboring the different TA systems were grown to mid-log phase before being placed over macrophage cell monolayers (5 × 10^5^ cells/well) in 24-well tissue culture plates at an infection ratio of approximately 10 bacteria per eukaryotic cell. After incubation at 37 °C for 45 min (time to internalization), infected cells were washed three times with PBS. Then, complete RPMI supplemented with 50 mg/mL gentamicin was added (2 h, 37 °C) to kill any remaining extracellular bacteria. Thereafter, the medium in the 24-well plates was replaced again with fresh complete RPMI supplemented with 5 mg/mL gentamicin. Host cells were then left in this medium for the remainder of the experiment to prevent extracellular growth of any released bacteria. At various time points following infection with *Salmonella*, the cells were washed three times with PBS, and bacteria were harvested by adding 300 µL 0.1% Triton X-100 solution to each well, for 3 min. Cell lysates were collected and serially diluted in PBS, plated on LB plates, and incubated O.N., after which bacterial CFUs were counted.

### 2.4. Chicken Experiments

All chicken experiments were carried out in accordance with the guidelines of the Israeli Ethics Committee (approval ID IL-17-1-43, 1 January 2017). Chickens were kept in designated isolation chambers and supplied with ad libitum food and water. Anesthetics were not required for any of the procedures performed in this study. Euthanasia for organ collection was performed with CO_2_.

### 2.5. In Vivo Activity of TA Systems and Challenge Experiments

**Figure 3:**
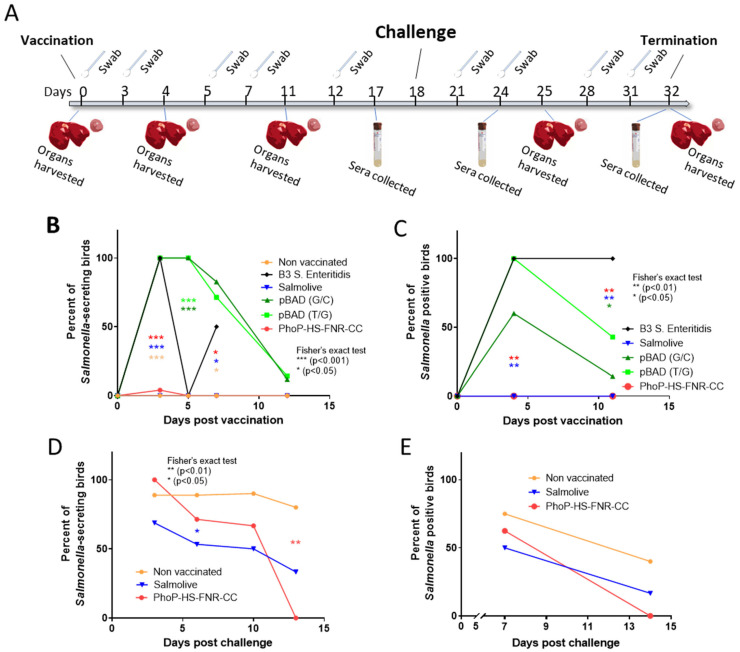
**Detection of *Salmonella* in cloacal swabs and internal organs following oral inoculation and challenge.** (**A**) One-day-old (day 0) chicks (n = 30) were inoculated with 2.5 × 10^8^ CFU *Salmonella* Enteritidis carrying pBAD-HS (G/C) (dark green), pBAD-HS (T/G) (light green), PhoP-HS-FNR-CC (red) TA systems, or the live attenuated vaccine Salmolive (blue). Inoculation of chicks with the wild-type *Salmonella* Enteritidis B3 strain (black) and water served as positive and negative controls (orange), respectively. Eighteen days after inoculation, chicks were challenged with WT *Salmonella* Enteritidis B3. Cloacal swab samples were collected on days 3, 5, 7, and 12 following inoculation and on days 3, 6, 10, and 13 post-challenge. Liver and spleen tissues were extracted from the sacrificed chicks on day 0 and then on days 4 and 11 following inoculation and on days 6 and 14 post-challenge. The percentages of (**B**) *Salmonella*-secreting birds and (**C**) birds with presence of *Salmonella* in their internal organs following inoculation was determined, as described in the Materials and Methods Section. Percentages of (**D**) *Salmonella*-secreting birds and (**E**) birds with presence of *Salmonella* in their internal organs following challenge was determined. Statistical significance for the pre-challenge tests (**B**,**C**) was determined by 2-tailed Fisher’s exact tests. Asterisks colored orange, blue, red, light green, and dark green signify statistical significance of results for the negative control, Salmolive, PhoP-HS-FNR-CC, pBAD-HS (T/G), and pBAD-HS (T/G) groups, respectively, as compared to the positive control. Statistical significance for post-challenge tests (**D**,**E**) was determined by 2-tailed Fisher’s exact tests. Asterisks colored blue and red signify statistical significance of results for the Salmolive and PhoP-HS-FNR-CC vaccine groups as compared to the negative control. *p* values are indicated on the graphs.

The in vivo experimental timeline is illustrated in Figure 3A. One-day-old specific pathogen-free (SPF) chicks were randomly divided into five groups with 30 birds per group. Chicks were orally inoculated with 2.5 × 10^8^ CFU of bacteria (in 0.5 mL). Groups 1 and 2 were inoculated with bacteria carrying pBAD-HS (G/C) or pBAD-HS (T/G), respectively, and group 3 was inoculated with bacteria carrying the combined PhoP-HS-FNR-CC TA systems. Group 4 was inoculated with wild-type *S.* Enteritidis B3 strain (positive control), group 5 was inoculated with water (negative control), and group 6 was inoculated with the live attenuated *S*. Enteritidis vaccine “Salmolive-E” according to the manufacturer’s instructions (Phibro). Each group was housed in separate isolators. Cloacal swabs were used to sample all birds on days 3, 5, 7, and 12 post-vaccination, and the presence of secreted *S.* Enteritidis was evaluated by plating swab samples on *Salmonella*-selective plates. On days 4 and 11 post-vaccination, livers and spleens of 4–7 birds from each group were removed, macerated. and plated on *Salmonella*-selective plates and then analyzed for the presence of *S.* Enteritidis. On day 18 post-vaccination, the non-vaccinated group and the groups vaccinated with Salmolive-E and bacteria carrying the combined PhoP-HS-FNR-CC TA system were challenged by wild-type *S.* Enteritidis B3 (2.5 × 10^8^ CFU in 0.5 mL). The presence of *Salmonella* in cloacal swabs was assessed on days 3, 6, 10, and 13 post-challenge, as described above. On days 7 and 14 post-challenge, livers and spleens of 6–10 birds from each group were removed and tested for the presence of *Salmonella*, as described above. Sera samples were collected from chicks in groups 3, 5, and 6, before challenge, on days 17 (N = 10, 16, and 14, respectively) 24 (6 days post-challenge; N = 10, 16, and 14, respectively) and 32 (14 days post-challenge; N = 4, 5, and 6, respectively) post-challenge, for measurement of IgY levels by enzyme-linked immunosorbent assay (ELISA). Samples (N = 4–6) from each group were further tested for serum IgA levels.

### 2.6. Enzyme-Linked Immunosorbent Assay (ELISA) for Testing Levels of SALMONELLA—Specific Antibodies

To quantitate serum levels of IgY and IgA raised against *Salmonella*, MaxiSorp ELISA plates (Thermo Fisher Scientific, Waltham, MA, USA) were coated (O.N. 4 °C) with 100 μL of formalin-killed wild-type *Salmonella* Enteritidis strain B3 (10^6^ CFU/mL), then blocked (5% skim milk with 0.05% tween 20, in PBS) for 2 h, at 37 °C. Plates were washed 3 times with 0.05% Tween 20 in PBS, after which, serially diluted (2-fold) sera samples were added and incubated for 1 h, at 37 °C. After rinsing the plates, rabbit anti-chicken IgY-peroxidase (Merck, Israel) or goat anti-chicken IgA-peroxidase (Merck) was added to the wells, according to the manufacturer’s recommendations, for 1 h at 37 °C. The plates were then rinsed, o-phenylenediamine dihydrochloride substrate was added, and absorption was measured at 450 nm. Antibody titers were determined as the last serum dilution in which absorption units value was 2-fold higher than that of the negative control (naïve serum).

### 2.7. Statistical Analysis

Graphs and statistical analyses were performed using GraphPad Prism software, version 9.3.1. The specific statistical tests used for each assay are indicated in figure legends.

## 3. Results

### 3.1. Effects of Attenuation of the Constitutive Hok Toxin Promoter on Salmonella Survival Under Restrictive Conditions and Within Macrophages

Attenuation of the Hok promoter in the pBAD-HS system significantly increased *Salmonella* survival in the absence of arabinose. Only 0.1% of the bacteria transformed with the pBAD-HS system survived 24 h of incubation under restrictive conditions as compared to growth in the presence of arabinose. In contrast, the attenuated pBAD-HS G/C and T/G constructs increased bacterial survival by more than 50- (5.1%) and 20-fold (2.1%), under the restrictive conditions, respectively (Figure 1B).

In macrophage tissue culture, *Salmonella* bearing the pBAD-HS G/C or T/G system survived for 20 h and 5 h, respectively, while the bacteria transformed with the pBAD-HS construct were completely eradicated within 2 h. (Figure 1C). In accordance with previously reported data [8], after 20 h, WT *Salmonella* displayed a 10-fold increase within macrophages (Figure 1C).

### 3.2. Survival of PhoP-HS-FNR-CC-Bearing Salmonella Under Restrictive Conditions and Within Macrophages

Under all tested restrictive conditions, *Salmonella* carrying the combined PhoP-HS-FNR-CC system (Figure 2A) demonstrated decreased survival compared to the control WT strain (Figure 2B). More specifically, as compared to the WT strain, bacterial propagation after 24 h under anaerobic conditions or di-cation (e.g., Ca^2+^ Mg^2+^) deprivation, was reduced by ~2.5 logs and ~1.5 logs, respectively (Figure 2B). Notably, under the combined restrictive conditions of both anaerobic conditions and di-cation deprivation, bacterial propagation was reduced by ~2.2 logs only, as the WT bacterial growth was inhibited as well. After 48 h in macrophage tissue culture, survival of *Salmonella* bearing the combined PhoP-HS-FNR-CC system was reduced to ~7.5% of the initial counts and by ~130-fold as compared to the WT, which continued to propagate (Figure 2C).

### 3.3. Survival and Shedding of Salmonella Carrying Modified TA Systems in Chickens

No adverse effects were observed in any of the vaccinated chicks. No bacteria were detected in the GI secretions of chickens inoculated with PhoP-HS-FNR-CC-bearing *S.* Enterica. Similar results were documented for chicks vaccinated with the negative control or the attenuated vaccine (“Salmolive-E”). In contrast, GI secretions of chicks vaccinated with bacteria carrying the pBAD-HS systems or with WT bacteria showed detectable bacteria throughout the 12-day experiment (Figure 3B). Furthermore, in both groups, bacterial penetration into internal organs was observed for up to 11 days, while no penetration occurred in the negative control groups or in chickens infected with the combined PhoP-HS-FNR-CC system or Salmolive-E (Figure 3C).

### 3.4. Challenge Experiments

As the combined PhoP-HS-FNR-CC system displayed high safety features, it was further tested in challenge experiments and compared to the commercial Salmolive-E vaccine. On day 3 post-challenge with the WT *Salmonella* B3 strain, high levels of secreted *Salmonella* were measured in all chick groups (Figure 3D). While WT bacteria were detected in secretions of the negative control group throughout the 12-day experiment, *Salmonella* levels in the group vaccinated with bacteria carrying the combined PhoP-HS-FNR-CC system continuously declined and reached undetectable levels between days 10 and 13. In contrast, 30% of birds vaccinated with Salmolive-E were still secreting *Salmonella* on day 13 (Figure 3D). After 14 days, no *Salmonella* was detected in the internal organs of PhoP-HS-FNR-CC-vaccinated chicks, while *Salmonella* was detected in the internal organs of ~16% and 40% of birds vaccinated with Salmolive-E and the negative control, respectively (Figure 3E).

### 3.5. Antibody Levels Following Immunization of Chickens with PhoP-HS-FNR-CC-Bearing Salmonella

**Figure 4:**
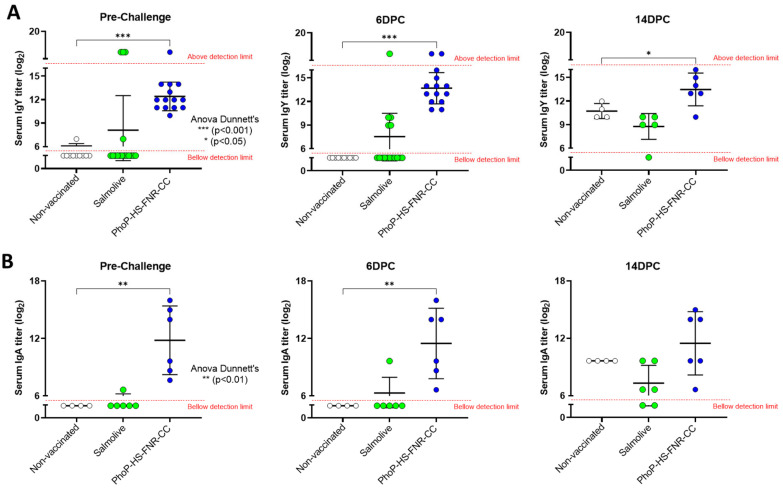
**Serum anti-*Salmonella* antibody levels following oral inoculation and challenge.** Sera samples were collected from chicks inoculated with water as a non-vaccinated negative control (white), 0.5 mL 2.5 × 10^8^ CFU *Salmonella* Enteritidis carrying the PhoP-HS+FNR-CC TA system (blue), or the live attenuated vaccine Salmolive (green), as detailed in Figure 3A. Enzyme-linked immunosorbent assays (ELISA) were performed to detect serum levels of IgY (**A**) and IgA (**B**) anti-*Salmonella* antibodies (see Section 2.5). Statistical significance as compared to the negative control was determined by ANOVA with Dunnett’s post hoc test.

Chicks immunized with *Salmonella* carrying the combined PhoP-HS-FNR-CC TA system developed significantly higher levels of Salmonella-specific IgY (mean titer: 12.4 (log_2_)) and IgA (mean titer: 11.8 (log_2_)) antibodies as compared to the non-vaccinated control. Notably, all chicks in the group vaccinated with PhoP-HS-FNR-CC-bearing *Salmonella* displayed high IgY (≥10 (log_2_)) and IgA (≥7.6 (log_2_)) antibody titers as compared to the group receiving the attenuated vaccine, in which 11 out of 16 chicks showed undetectable IgY (≤6 (log_2_)) titers and only 1 chick displayed detectable low IgA titer (6.6 (log_2_)) (Figure 4).

No significant changes in antibody levels were observed 6 days after challenge with the virulent WT Salmonella B3. As expected, 14 days following challenge, elevations of both IgY (Figure 4A) and IgA (Figure 4B) levels were observed in the non-vaccinated group. No significant elevation in antibody levels was observed in groups vaccinated with the attenuated vaccine or with *Salmonella* carrying the combined TA system (Figure 4).

## 4. Discussion

*Salmonella* vaccines for poultry requires delicate balance between immunogenicity and safety. Inactivated vaccines are regarded as highly safe; however, the immune response they induce is often less effective for protection [4,21]. In addition, inactivated vaccines necessitate the addition of immune stimulators, as well as individual injection to each animal, rendering them impractical for vaccination in large flocks. The commonly used attenuated bacterial vaccines may be administered systemically (e.g., via drinking water); however, safety challenges such as reversion to virulence and increased risk of environmental spread may pose concern [4,22,23].

In addition, development of attenuated strains is a long laborious process, and their efficiency can rapidly decline due to incompatibility with circulating field strains that continue to evolve [4,24,25,26].

In order to overcome current limitations and achieve high vaccine safety together with robust immunogenicity, we developed a novel adaptable vaccination platform utilizing self-destructive live virulent *Salmonella* Enteritidis driven by TA systems. The proposed TA-based platform does not involve genomic manipulation and may be incorporated with relative ease into newly emerging wild-type *Salmonella* Enteritidis isolates to enable rapid development of field-compatible, orally administered, safe, and efficacious vaccines.

As previously described by Gruzdev et al., the constructs incorporating several TAs led to self-destruction of virulent *Salmonella* Enteritidis both in vitro and in vivo in inoculated animals within a period that was sufficient for the induction of a protective immune response [8]. The present study focused on optimizing the tradeoff between safety and immunogenicity of two TA systems. More specifically, the work set out to enhance immunogenicity of the host-independent activation of pBAD-HS, which previously displayed high safety together with low immunogenicity by attenuation of its Hok toxin promoter. At the same time, it attempted to enhance the safety of the immunogenic host-dependent FNR-CC system through its combination with the PhoP-HS TA system.

In the pBAD-HS system, attenuation of the Hok toxin promoter to reduce toxin expression significantly increased bacterial survival in the absence of arabinose, indicating successful modulation of the TA system activity. The modification also prolonged bacterial viability in macrophages as compared to the original promoter-induced system, potentially enhancing the window for immune stimulation. However, in the chickens, the attenuated Hok toxin promoter enabled bacterial survival at levels comparable to those of the WT strain. These results highlight the potential for fine-tuning bacterial persistence by manipulating promoter strength, which is crucial for optimizing vaccine efficacy and safety. Alternative manipulation of the promoter may achieve the desired balance between the stimulation duration and safety features of this vaccine.

Upon reaching the small intestine, *Salmonella* invades intestinal epithelial cells. Following *Salmonella* invasion, it can be internalized by macrophages, which are unable to kill it, as the bacteria can inhibit the fusion of phagosomes with secondary lysosomes [27]. Gruzdev et al. [8] previously reported that the FNR-CC system, when implemented alone, was not activated following uptake by macrophages, resulting in bacterial survival rates similar to those of the WT control strain. In contrast, the combined PhoP-HS-FNR-CC system resulted in decreased *Salmonella* survival under restrictive conditions of low oxygen and low calcium/magnesium mimicking environmental conditions present in the small intestine and within macrophages, respectively. A continuous reduction in CFU counts of bacteria bearing the combined system was measured, reaching more than 90% reduction in survival 48 h post-internalization. Taken together, the combination of both TA systems activated by two separate environmental conditions present in the host may serve as a two-step verification system to enhance vaccine safety by ensuring the destruction and clearance of *Salmonella* carrying TA systems.

In in vivo experiments, both the commercial vaccine and the *Salmonella* carrying the PhoP-HS-FNR-CC system were undetected in cloacal swabs and internal organs by day 5 following inoculation, demonstrating complete clearance of the vaccine bacteria and underscoring their enhanced safety profile. However, the challenge experiments suggest the potential of the combined TA system to outperform the commercial attenuated vaccine, as the challenge strain in the chicken vaccinated with the *Salmonella* carrying PhoP-HS-FNR-CC system was completely eradicated within 14 days, while in some chickens immunized with the commercial vaccine, *Salmonella* was still detected. In parallel, both pre- and post-challenge, higher levels of anti-*Salmonella* IgY and IgA antibodies were measured in the serum of chickens vaccinated with the combined PhoP-HS-FNR-CC system as compared to the group vaccinated with the live attenuated vaccine. A possible explanation for these high antibody levels detected may arise from the subsequent release of intracellular bacterial immune-enhancing molecules following cell-induced lysis.

Notably, in chicks vaccinated with the commercial vaccine, some reduction of *Salmonella* was observed in the absence of elevated antibody levels, suggesting that cellular immunity may also be activated.

Further studies will be necessary to investigate the duration and breadth of protection conferred by this vaccine approach. In addition, TA-based *Salmonella* vaccines can also be considered to serve as a vector for subunit protein vaccines, similarly to previously reported works [28,29,30,31,32,33].

## 5. Conclusions

This study demonstrates the feasibility of using TA-based systems inserted into wild-type *Salmonella* enteritidis for safe and efficacious vaccination. It highlights the ability of tuning both host-dependent and host-independent TA systems to either enhance or reduce bacterial persistence, aiming to optimize vaccine safety and efficacy.

Such a platform can potentially be used to rapidly update and produce vaccines upon the appearance of newly emerging circulating strains of *Salmonella* enteritidis capable of evading outdated vaccines.

Overall, the ability of *Salmonella* carrying the PhoP-HS-FNR-CC system to provide protection against challenge with wild-type *Salmonella* demonstrates the potential of the TA-based vaccination platform to induce protective immunity against *Salmonella* infection in poultry.

## Figures and Tables

**Table 1:** Growth conditions for bacterial strains carrying different stimulation systems.

TA System	Permissive GC	Restrictive GC
pBAD-HS pBAD-HS (G/C) pBAD-HS (T/G)	LB + arabinose (0.2%)	LB
PhoP-HS-FNR-CC	LB	1. TGB #
		2. LB + EDTA (2 mM)
		3. TGB # + EDTA (2 mM)

# Without aeration. TA: toxin/antitoxin, CC: CeaB/CeiB, HS: Hok/Sok, GC: growth conditions, TGB: thioglycolate broth, LB: Luria broth.

## Data Availability

Data will be available upon request.
